# Methylation Profile of Single Hepatocytes Derived from Hepatitis B Virus-Related Hepatocellular Carcinoma

**DOI:** 10.1371/journal.pone.0019862

**Published:** 2011-05-23

**Authors:** Ran Tao, Jun Li, Jiaojiao Xin, Jian Wu, Jing Guo, Liyuan Zhang, Longyan Jiang, Wu Zhang, Zhe Yang, Lanjuan Li

**Affiliations:** 1 State Key Laboratory for Diagnosis and Treatment of Infectious Diseases, School of Medicine, First Affiliated Hospital, Zhejiang University, Hangzhou, Zhejiang, China; 2 Department of Surgery, School of Medicine, First Affiliated Hospital, Zhejiang University, Hangzhou, Zhejiang, China; The University of Kansas Medical Center, United States of America

## Abstract

**Background:**

With the development of high-throughput screening, a variety of genetic alterations has been found in hepatocellular carcinoma (HCC). Although previous studies on HCC methylation profiles have focused on liver tissue, studies using isolated hepatocytes are rare. The heterogeneity of liver composition may impact the genuine methylation status of HCC; therefore, it is important to clarify the methylation profile of hepatocytes to aid in understanding the process of tumorigenesis.

**Methods and Findings:**

The global methylation profile of single hepatocytes isolated from liver tissue of hepatitis B virus (HBV) related HCC (HBHC) was analyzed using Illumina Infinium Human Methylation27 BeadChips, and combined bisulfite restriction analysis (COBRA) and bisulfite sequencing were used to validate the 20 significant hypermethylated genes identified. In this study, we found many noteworthy differences in the genome-wide methylation profiles of single hepatocytes of HBHC. Unsupervised hierarchical clustering analysis showed that hepatocyte methylation profiles could be classified according to three cell types: hepatocytes of HCC, adjacent hepatocytes and normal hepatocytes. Among the 20 most hypermethylated genes in the hepatocytes of HBHC, 7 novel genes (WNK2, EMILIN2, TLX3, TM6SF1, TRIM58, HIST1H4Fand GRASP) were found to be hypermethylated in HBHC and hypomethylated in paired adjacent liver tissues; these findings have not been reported in previous studies on tissue samples.

**Conclusion:**

The genome-wide methylation profile of purified single hepatocytes of HBHC was aided in understanding the process of tumorigenesis, and a series of novel methylated genes found in this study have the potential to be biomarkers for the diagnosis and prognosis of HBHC.

## Introduction

Hepatocellular carcinoma (HCC) is one of the most common cancers in the world and is commonly developed from liver cirrhosis that are secondary to viral infection [1]. China is one of the highest prevalent areas of HCC as chronic hepatitis B carriers account for more than 10% of its population. The prognosis of HCC patients is poor even after partial hepatectomy due to the high rates of metastasis and relapse [2], [3], and there is an urgent need to elucidate the mechanism of hepatocarcinogenesis.

Epigenetic changes have been reported recently as one of the mechanisms of tumorigenesis, and DNA methylation, which is one of the major epigenetic alterations, has also been reported in HCC. Aberrant DNA methylation of promoter CpG islands has been associated with global hypomethylation and specific loci hypermethylation, which are believed to have potential as diagnostic markers in the progression of malignant tumors. In HCC, DNA hypermethylation of candidate genes, including GSTP1 [4], RASSF1A [5], RIZ1, APC [6], SOCS1 [7], APC [8], and E-cadherin[9], has been examined using methylation sensitive polymerase chain reaction, combined bisulfite restriction analysis (COBRA), and bisulfite sequencing techniques in previous studies. With the development of genome-wide screening, the global methylation profiling of cancer has become clearer than ever before. A group of potential tumor biomarker genes has been reported in HCC [10]. Different gene methylation profiles have been found in HCCs with different etiological backgrounds [11], [12], [13], pathophysiological processes [10], and histological characteristics.

However, most of the large-scale screening profiles have been performed on tissue samples. Tissues often consist of a heterogeneous population of cells. The same is also true in cancerous tissues. In addition to tumor cells, HCC tissues can have fibroblasts, stromal cells, vasculature, inflammatory cells and other non-parenchymal cells [14]. Although these non-parenchymal cells have some effect on the progression of cancer, this mixed population may distort or even hide the genuine methylation status of cancer cells, making diagnosis difficult to determine and experimental results hard to interpret.

Several studies from independent labs have reported gene expression and proteomic profile changes in isolated cells compared to primary tissues. Harrell et al. compared the gene expression of whole organs to isolated cells from the organ and showed a distinctly different gene expression profile, with only minor overlap between the two samples [15]. Waanders et al. reported that the proteomic analysis of single pancreatic islets was different from that of pancreatic tissue, suggesting that single-cell analysis is more readily accessible to proteomic measurements [16]. Even the tumor epithelium and tumor-associated stroma have shown different methylation profiles, as GSTP1 and RARbeta2 were found to be hypermethylated in the tumor epithelium and less so in tumor-associated stroma [17]. In the context of the liver, hepatocytes from HCC have shown reduced heterogeneity and contamination in tissue samples and present less protein spots than HCC tissue [18]. Considering the difference between isolated hepatocytes and liver tissue, the gene expression and proteomic profiles of hepatocytes isolated from cirrhotic nodules [19], fetal livers [20], HCC lymphatic metastases [21] and neoplasms [18] have been studied. The analysis of these hepatocytes has revealed not only specific patterns that may reflect the prognosis and drug sensitivity of cancer cell [22], [23] but also the identity of genes involved in malignant transformation, progression [24], and tumor metastasis [21]. The homogeneity of isolated hepatocytes may allow the elucidation of the mechanism of tumorigenesis in HCC, with the global genome-wide methylation profile of HCC cells being of particular interest.

In the present study, we used a four-step collagenase perfusion method to separate hepatocytes from liver tissues and analyzed the aberrant promoter methylation of three paired liver cancer samples and adjacent liver samples using Illumina Infinium Human Methylation27 BeadChips that contained 27,000 CpGs. And to eliminate the effect of different clinical backgrounds, we focused on HBV related HCC (HBHC). The CpG island methylation profile of hepatocytes isolated from primary tissues and distinct pathways in single hepatocytes from HBHC were obtained and revealed a group of potential novel biomarkers in HBHC.

## Results

### Isolation and characterization of separated cells from liver tissues

For clarification, in this study, the hepatocytes separated from live cancer tissues were called HHCs, while HAs were those that were collected from adjacent non-cancerous liver tissues and HNs were those that were isolated from normal liver tissues. The isolated hepatocyte motility rate was similar in the three groups (95%±4%) using a four-step collagenase perfusion method; therefore, a cell motility rate >90% was chosen for further analysis. The total number of separated hepatocytes was 1.38±0.38×106 per gram of hepatic tissue, and the contamination rate of non-parenchymal cells in the HHC population was <2%, with a small cellular fraction as determined using microscopic analysis.

### Genome-wide DNA methylation profiling identifies methylated genes in freshly isolated hepatocytes and liver tissues

**Figure 1:**
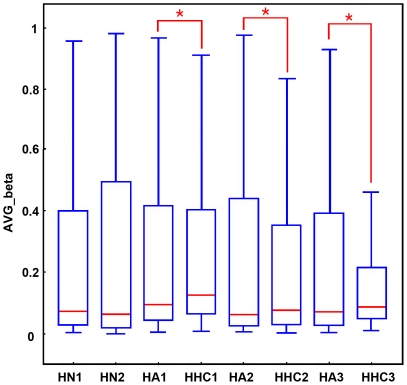
Average promoter methylation of single hepatocyte samples measured by BeadChip. Average promoter methylation of hepatocyte of normal liver (HN), adjacent liver (HA) and HCC (HHC) for all the 27,000 CpG loci. The illustrative box plots represent the mean, 25th percentile, 75th percentile and range of the data. Significant differences (P<0.05) between tumor and surrounding tissue are represented with an asterisk (*).

Global DNA methylation profiles were measured using Illumina Infinium Human Methylation27 BeadChips, which target 14,475 total refseq genes, 12,833 well-annotated genes described in the NCBI CCDS database, 144 methylation hotspots in cancer genes, 982 cancer-related targets and 110 miRNA promoters. Three pairs of HHCs and HAs were included for comparison using the HNs as the control. Using this approach, the average methylation rate of HHCs was higher than that of matched HAs (Figure 1) as reported in previous study[25].

**Figure 2:**
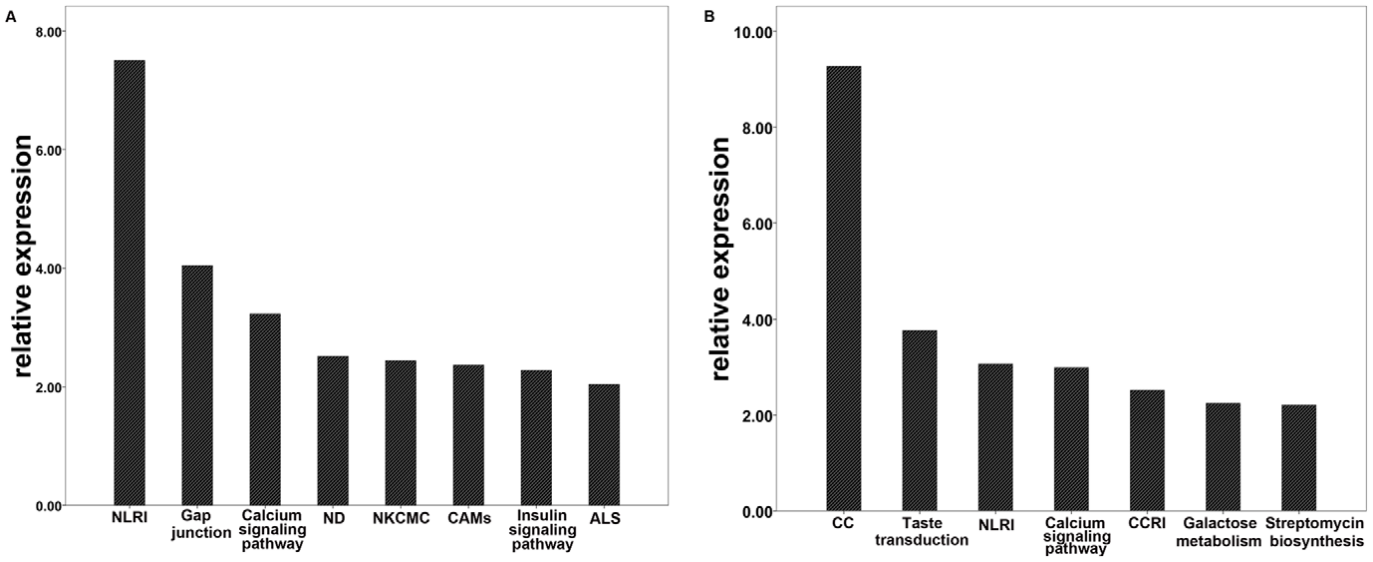
Pathway analysis of single hepatocytes derived from HBHC. Significantly aberrant methylated gene (P<0.05) in HHC enriched in KEGG pathway are summarized in A (hypermethylation) B (hypomethylation), NLRI, Neuroactive ligand-receptor interaction; ND, Neurodegenerative Disorders; NKCMC, Natural killer cell mediated cytotoxicity; CAMs, Cell adhesion molecules;ALS, Amyotrophic lateral sclerosis; CC, Cell Communication.

To distinguish the genes that were differentially methylated in HHCs, methylated genes were filtered according to P<0.05 and a delta β>0.15. HHCs showed 792 genes that were hypermethylated and 533 genes that were hypomethylated compared with HNs, and 345 and 796 genes were hypermethylated and hypomethylated, when compared with HAs respectively. When compared to both HAs and HNs combined, HHCs showed 315 and 460 genes that were hypermethylated and hypomethylated, respectively. KEGG (Kyoto Encyclopedia of Genes and Genomes) analyses were performed to identify the pathways with which the aberrant genes in HHCs were associated. Hypermethylated genes in HHCs were enriched in the KEGG pathway, most of which have been reported to be involved in the following cancer pathways: gap junction (p = 9.1×10-5), calcium signaling (p = 5.8×10-4), natural killer cell-mediated cytotoxicity (p = 3.6×10-3), cell adhesion molecules (p = 4.3×10-3), and apoptosis (p = 0.0322). Hypomethylated genes were enriched in cell communication (p = 5.3×10-10) and calcium signaling (p = 1.02×10-3; Tables 1, 2 and Figure 2) pathways.

**Table 1:** Hypermethylated gene in single hepatocytes from HBHC enriched in KEGG pathway.

KEGG Pathway	Gene Number	Gene Symbol	Enrichment	P Value
Neuroactive ligand-receptor interaction	15	GRP83, CRHR2, ADRA2B, DAB2IP, ADRB3, DRD4, EDNRB, F2R, GALR1, NPBWR1, HTR7, NMBR, NPY, VIPR2, ALDH1A2	O = 15;E = 2.4491;R = 6.1247;P = 3.13e-8	3.13E-08
Gap junction	6	ADCY5, DAB2IP, ADCY4, CUCY1A2, CX36, TUBB6	O = 6;E = 0.7253;R = 8.2724;P = 9.10e-5	9.10E-05
Calcium signaling pathway	7	DAB2IP, ADRB3, EDNRB, ADCY4, ERBB4, F2R, HTR7	O = 7;E = 1.4165;R = 4.9418;P = 5.85e-4	5.85E-04
Neurodegenerative Disorders	3	NFH, NGFR, UCHL1	O = 3;E = 0.2901;R = 10.3413;P = 3.03e-3	3.03E-03
Natural killer cell mediated cytotoxicity	5	VAV3, HLA-G, PIK3CD, SYK, TNFRS10F	O = 5;E = 1.0155;R = 4.9237;P = 3.62e-3	3.62E-03
Cell adhesion molecules (CAMs)	5	CNTNAP2, HLA-G, ITGA9, ITGB8, CD8A	O = 5;E = 1.0581;R = 4.7255;P = 4.31e-3	4.31E-03
Insulin signaling pathway	5	MKNK2, PFKP, PIK3CD, PRKAR1B, IRS4	O = 5;E = 1.1093;R = 4.5073;P = 5.26e-3	5.26E-03
Amyotrophic lateral sclerosis (ALS)	2	NEF3, NEFHG	O = 2;E = 0.1451;R = 13.7836;P = 9.07e-3	9.07E-03

O is the observed gene number in the pathway. E is the expected gene number in the pathway (Expected number of genes in a specific pathway for an interesting gene set  =  Total number of genes in the pathway for the reference set × Total number of genes in the interesting set/Total number of genes in the reference set). R is the ration of enrichment for the pathway (R = O/E). P is the p value indicating the significance of enrichment calculated from hypergeometric test. It is given for the pathways with R>1.

**Table 2:** Hypomethylated gene in single hepatoytes from HBHC enriched in KEGG pathway.

KEGG Pathway	Gene Number	Gene Symbol	Enrichment	P Value
Cell Communication	13	LAMC3, GJB4, CX40.1, K6IRS3, KRT1, KRT5, KRTHA1, KRTHA3A, KRTHA3B, KRTHA4, KRTHA5, KRTHB4	O = 13;E = 1.2773;R = 10.1777;P = 5.35e-10	5.35E-10
Taste transduction	5	GNAS, GRM4, TRPM5, ACCN1, TAS2R8	O = 5;E = 0.5231;R = 9.5584;P = 1.71e-4	1.71E-04
Neuroactive ligand-receptor interaction	11	CYSLTR1, GABRA6, GHRH, NPBWR2, GRIK5, GRM4, GRM8, HRH2, MC3R, NTSR1, NMUR2	O = 11;E = 3.4912;R = 3.1508;P = 8.54e-4	8.54E-04
Calcium signaling pathway	8	CYSLTR1, GNAL, GNAS, HRH2, NOS3, NTSR1, ATP2B3, SLC8A1	O = 8;E = 2.0193;R = 3.9618;P = 1.02e-3	1.02E-03
Cytokine-cytokine receptor interaction	9	IL17B, IL28B, XCR1, HGF, CCL7, CCL13, CCL18, TPO, TNFRSF10A	O = 9;E = 2.9438;R = 3.0573;P = 3.02e-3	3.02E-03
Galactose metabolism	3	HK2, HK3, LALBA	O = 3;E = 0.3649;R = 8.2214;P = 5.69e-3	5.69E-03
Streptomycin biosynthesis	2	HK2, HK3	O = 2;E = 0.1216;R = 16.4474;P = 6.23e-3	6.23E-03

**Figure 3:**
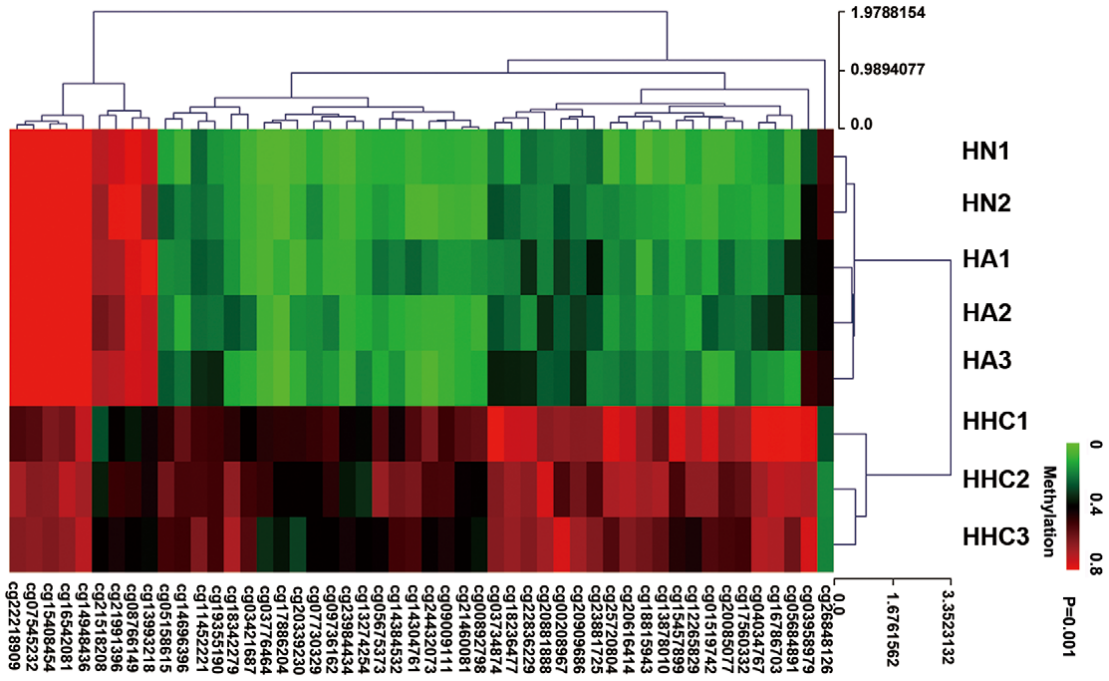
Heat map of methylated gene in the Illumina Infinium methylation assay. Heat map of 64 CpG locus were different methylated in 3 pairs of hepatocyte from HBHC(HHC) and adjacent liver(HA) with 2 normal liver(HN) as control(p<0.001). The hypermethylated and hypomethylated genes were indicated by red and green respectively. Unsupervised hierarchical cluster analysis represents a significent distinguishing HHCs from HAs and HNs. The Euclidean distance between two groups of samples is calculated by the average linkage measure between members of the two groups concerned.

We further restricted inclusion criteria as P<0.001 and the delta β>0.15, which resulted in the identification of 63 CpG sites corresponding to 62 genes (Table S1); the average distance from the 63 CpG sites to the transcription start site (TSS) was 256.58 bp. A total of 50 CpG sites showed significantly higher methylation in HHCs relative to the other two groups of hepatocytes. While 10 CpG sites outside of the CpG islands showed less methylation in HHCs, there were only 2 CpG sites inside CpG islands that showed hypomethylation. A total of 45 CpG sites exhibited a significant monotonic decreasing trend in the methylation rate (HHCs>HAs>HNs), while 8 CpG sites showed increasing trends (HHCs<HAs<HNs). Among the 63 genes showing the most significant differences in methylation, 38 genes have been associated with cancer in previous studies. Among them, 10 genes have been reported in HCC, and ZMYND10 [26], SYK [27], DAB2IP [28], and DLEC1 [29] have been reported to be tumor-suppressor genes in HCC. Unsupervised hierarchical clustering analysis with a P<0.001 showed that in the single hepatocytes group, the cells could be classified into three types: HN, HA, HHC (Figure 3).

The methylation profile in HAs was also compared with that of HNs, and 124 and 115 genes were identified as differently methylated (p<0.05) and hypermethylated, respectively. A number of these genes were associated with MAPK (FGF2, BDNF, TGFB3, FGF23, and FGF17) and colorectal cancer (dcc, cycs, and tgfb3) signaling pathway.

### Methylation analysis in paired normal and liver cancer tissues

**Figure 4:**
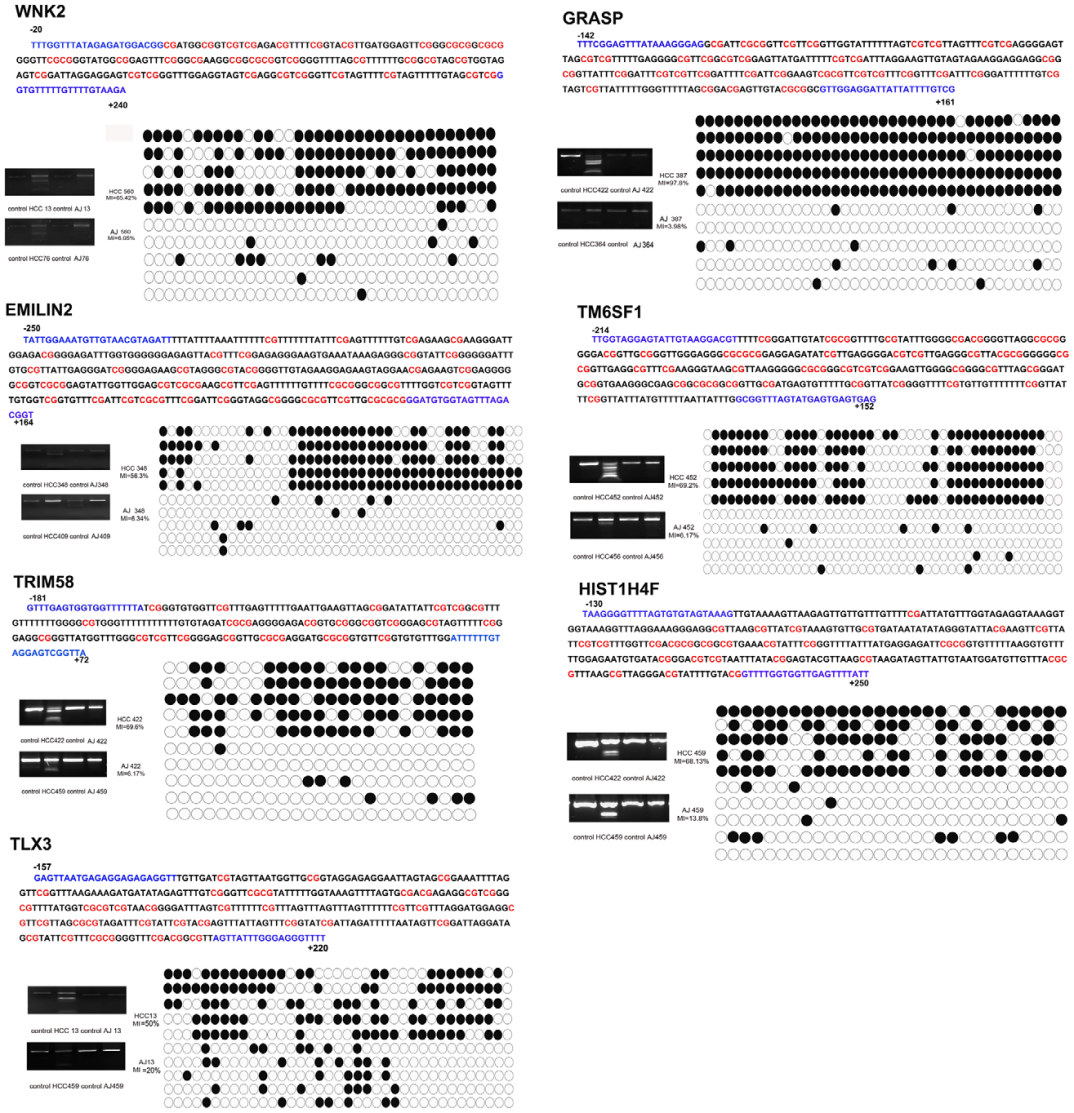
DNA methylation status of seven selected genes in HBHC and adjacent liver tissue. COBRA analysis were performed as amplified PCR of sodium bisulfite-converted genomic DNAs, PCR products incubated with BstUI to measure the methylation rate and samples without incubation with BstUI were listed as control. Selected PCR products were cloned into T-A clones and sequencing. Five samples of each alleles listed were shown by black and white circles represent methylated and unmethylated CpG dinucleotides respectively. "MI" was the abbreviation of methylation index, calculated as the percentage of the methylated dinucleotides versus total. Detail methylated sites were marked in red, the position of COBRA primers is also shown in blue, covering the region was respect to the transcription start site. AJ was the abbreviation of adjacent liver tissue.

To further investigate whether the methylation profiles of isolated hepatocytes were consistent with tissue samples, we chose the 20 most significantly hypermethylated genes in five pairs of HBHC tissues and adjacent liver tissues for further validation using COBRA. Two previously reported genes (ZMYND10 and SYK) and one unknown gene (DKFZp434I1020) were excluded from the analysis. The PCR products of these seventeen genes amplified from bisulfite-treated cancer and adjacent tissues were digested using the methylation-sensitive restriction enzyme BstUI. Two of the genes (KIF17 and ATP8A2) showed no methylation in either cancer or matched tissues, and eight genes (EPHA4, OVOL1, UTF1, ADAM8, BOLL, FOXE3, GUCY1A2, and ADCY5) were equally methylated in the two groups. These ten genes were removed from further analysis, and the remaining seven genes were analyzed in ten paired liver tissues. These genes were frequently methylated in HBHC, while those in the matched non-cancerous liver tissues were either methylated to a significantly lower extent or not methylated at all. A summary of the promoter methylation analysis is shown in Tables 3 and 4 and the cloning and sequencing analysis results are shown in Figure 4. These results indicate that cancer tissues have much higher methylation rates than the related non-cancerous tissues, which confirm the results from COBRA.

**Table 3:** COBRA analysis of 17 selected genes in primary tumor and adjacent liver.

Genes	Primary Tumor	Adjacent Liver	P Value
**WNK2**	3/5	0/5	0.038
**FOXE3**	5/5	4/5	0.292
**GUCY1A2**	2/5	3/5	0.294
**EMILIN2**	3/5	0/5	0.038
**TRIM58**	3/5	0/5	0.038
**ADCY5**	4/5	2/5	0.197
**OVAL1**	3/5	2/5	0.527
**ADAM8**	5/5	4/5	0.292
**GRASP**	3/5	0/5	0.038
**UTF1**	4/5	4/5	1
**BOLL**	2/5	5/5	0.079
**TM6SF1**	4/5	0/5	0.01
**KIF17**	0/5	0/5	1
**EPHA4**	1/5	0/5	0.292
**ATP8A2**	3/5	3/5	1
**HIST1H4F**	3/5	0/5	0.038
**TLX3**	3/5	0/5	0.038

The genes selected from Illumina Infinium methylation assay were validated in COBRA with five pairs primary tumor and adjacent liver, ten genes shared the same methylation rate in primary and adjacent liver by our COBRA primers were excluded.

**Table 4:** COBRA analysis of 7 selected genes in primary tumor and adjacent liver.

Genes	Primary Tumor	Adjacent Liver	P Value
**WNK2**	7/10	2/10	0.025
**EMILIN2**	7/10	0/10	0.001
**TRIM58**	7/10	1/10	0.006
**GRASP**	7/10	2/10	0.025
**TM6SF1**	8/10	2/10	0.005
**HIST1H4F**	7/10	0/10	0.001
**TLX3**	6/10	1/10	0.02

Seven genes confirmed frequent hypermethylation in primary tumor tissues (p<0.05) were selected for further analysis in ten pairs of primary tumor and adjacent liver tissues, frequent hypermethylation (P<0.05) were observed in these genes.

### Expression analysis in cell lines with 5-aza-dc

**Figure 5:**
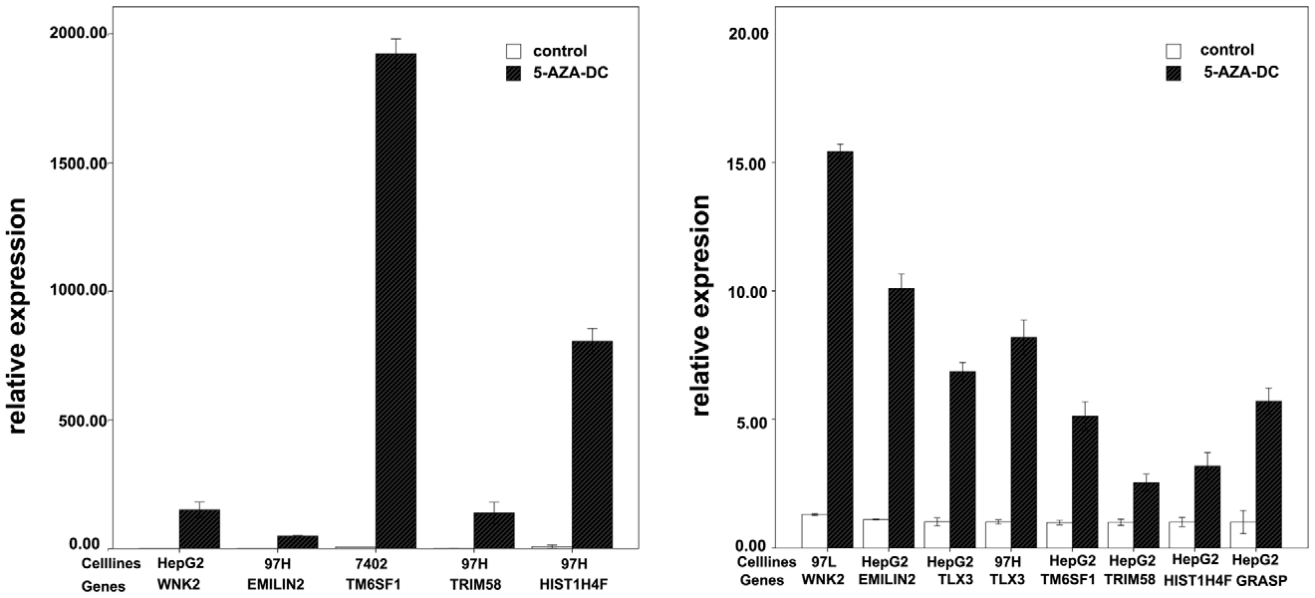
Gene re-expression after treated with 5-aza-dc. Gene expression level was analyzed by quantitative reverse transcription-polymerase chain reaction. All selected genes were unregulated after treated with 10 uM 5-aza-dcC at least in one cell lines, the expression of cell lines before 5-aza-dc treatment was analyzed as control.

To confirm the role of methylation in gene expression silencing, we treated four hepatoma cell lines with the demethylating agent 5-aza-dc. Seven genes that were validated in ten paired HBHC and adjacent liver tissues using COBRA were selected for this experiment, and their expression was analyzed using real-time quantitative polymerase chain reaction (RT-PCR). All of the selected genes were upregulated after 5-aza-dc treatment in at least one cell line (Figure 5), and one gene, TLX3, was upregulated in all four cell lines. The results are summarized in Table 5.

**Table 5:** Seven selected genes were analyzed in four cell lines by RT-PCR.

Genes	HepG2	7402	97L	97H
**WNK2**	151	0.68	1.38	0.086
**EMILIN2**	9.4	0.66	0.28	48.8
**TLX3**	6.8	1.3	6.8	8.02
**TM6SF1**	5.18	352.8	4.9	(-)
**TRIM58**	2.55	0.3	0.03	107.8
**HIST1H4F**	3.2	0.64	0.004	100.7
**GRASP**	5.66	0.65	0.05	(-)

The expression of the selected genes after treated with 5-aza-dc was summarized in the table 5 and before treatment was used as control. The relative expression rate  =  the expression of gene after treatment/the expression of gene before treatment.

## Discussion

Carcinogenesis is a multi-stage, multi-mechanistic process and the details of the progression of a normal cell as it transforms into a cancer that exhibits excessive proliferation and reduced apoptotic potential are still unclear. The global methylation profile helps us to clarify the progression of hepatocarcinogenesis. In this study, we analyzed the methylation profiles of single hepatocyte isolated from HBHC using Illumina Infinium Human Methylation27 BeadChips to better understand the molecular mechanisms underlying tumorigenesis.

The collagenase perfusion method is a classic method to isolate hepatocytes from liver tissues. This method were used to isolated hepatocyte from normal liver[30], [31], liver cirrhosis[32] and HCC[20], [22], [23], [33] to study molecular mechanisms. Fibrous liver connective tissue was removed through successive perfusion with EDTA, dispase, collagenase type IV, and DNase I. Centrifuged at low speed to eliminate non-parenchymal cells. Single isolated hepatocyte is not only different from liver tissue without the latter's heterogeneity which might influence the methylation profile [13], but is also different from cell lines. Because cell lines were cultured in vitro and did not have the background of chronic viral infection, hypermethylation in cell lines could be a background event caused by repeated passages and the culture environment, which could be significantly different from primary malignancies [34]. Therefore, we focused our study on freshly isolated hepatocytes, which not only exhibit the pathology background but are also homogeneous.

Using the isolated single hepatocytes from HBHC, we detected novel genes that were specifically methylated. However, among these genes, only a few overlapped with previous studies. Beside the differences between single hepatocytes and liver tissue, one explanation for these differences is that the studies varied in pathogen, pathology, tumor grade, race and other clinical data. In this study, we selected all samples according to the same criteria to eliminate the effect of different clinical backgrounds. In addition, all HCC specimens were moderately or poor differentiated, and all patients suffered from hepatitis B virus (HBV) and exhibited the same underlying cirrhosis etiology. Another explanation for the variability of the findings from the multiple studies could be that the methylation microarrays from each company detected methylated genes in the human genome using different detection methods with variations in the sensitivity of detection and filter range.

In this study, the HHC methylation profile detected genes that were linked with several cancer-associated pathways. Gap junction genes (ADCY5, DAB2IP, ADCY4 CUCY1A2, CX36, and TUBB6) were significantly hypermethylated in HHCs (p = 0.000091) and have shown significantly reduced expression in HCC compared with normal livers in previous studies [35] [31]. Cancer was one of the first pathologies to be associated with gap junction defects, and tumor formation, invasiveness and cancer cell dissemination have all been closely correlated with gap junctions[36]. The inhibition of gap junctional intercellular communication (GJIC) results in a block in cell communication and the loss of the control of endocrine regulation between normal cells to regulate growth and terminally differentiate, thus resulting in immortalization and escape from apoptosis [37], [38]. GJIC is postulated to be an important mechanism to control growth and differentiation and eliminate the interference from non-parenchymal liver cells, and it was significantly associated with isolated hepatocyte methylation profiles.

Calcium signaling, cell adhesion molecules and apoptosis are all closely related to gap junctions. Calcium signaling represents a general mechanism of intercellular signaling via the gap junction-mediated diffusion of Ca2+/inositol 1,4,5-triphosphate [37]; cancer cell transformation, cell proliferation and cell death are associated with a major rearrangement of Ca2+ pumps, Na/Ca exchangers and Ca2+ channels, which leads to enhanced proliferation and an impaired ability to die that ultimately determines cell fate [38]. Cell adhesion molecules play an important role in regulating cell survival, proliferation and motility [39], and apoptosis is an important link between inflammation and liver cancer. Inflammatory hepatitis might activate NF-κB by the concerted action of cytokines that are resistant to apoptosis and promote cell survival of pre-cancerous hepatocytes [27]. Another pathway that is associated with cancer is natural killer cell-mediated cytotoxicity. Nature killer cell(NK cell) play a role in mediating innate host immune responses, which are regulated by a balance between activating and inhibitory receptors. The dysregulation of natural killer cell-mediated cytotoxicity breaks this balance because upregulation of the ligands for inhibitory receptors and/or the loss of ligands for activating receptors aids in tumor cell escape from NK cell recognition [40].

Except the distinct pathways identified in HHCs, a group of aberrantly methylated genes was selected. EPHA4, KIF17, OVOL1, UTF1, ADAM8 BOLL, FOXE3, GUCY1A2, and ADCY5 exhibited the same promoter methylation status in HBHC and adjacent liver tissue, suggesting that the HHC methylation is acquired during cirrhosis and chronic inflammation with persistent viral infection during the pre-cancerous state before the formation of the tumor. SYK and ZMYND10 (also known as BLU) were hypermethylated in HHCs in our studies. These genes have been reported to be methylated in HCC, indicative of tumor-suppressor genes. SYK has been highly correlated with lower overall survival rates. [26], [27]. WNK2, emilin2, tlx3, tm6sf1, trim58, hist1h4f, and grasp were significantly hypermethylated in HBHC. Wnk2 belongs to a serine/threonine kinase subfamily that has the catalytic lysine in a non-canonical position. WNK2 point mutations have been found in lung cancer, adult glial tumors and other solid tumor types, and it has also been reported to be a tumor suppressor [41]. EMILIN2 belongs to a family of extracellular matrix glycoproteins that contain an EMI domain at the N-terminus and a C1q domain at the C-terminus. EMILIN2 has been shown to suppress the growth of breast and other cancer cells and trigger apoptosis in cancer cells via the extrinsic apoptotic pathway following EMILIN2 binding to the trail receptors DR4 and DR5 [42]. TLX3 has been shown to be hypermethylated in prostate cancer [43]. Tm6sf1, trim58, hist1h4f, and grasp were identified as novel hypermethylated genes in HBHC; these genes were methylated in HHCs and HBHC tissues, suggesting that the methylation of these genes might have critical role in tumorigenesis and cancer progression. These novel genes were upregulated in response to 5-aza-dc in at least one of the tested cell lines, and based on the different invasive behaviors of these cell lines, the low expression was regulated by hypermethylation.

Another observation is the distinct methylation profile of isolated hepatocytes from non-cancerous livers, which was different from that of normal hepatocytes, as reported in previous studies with tissue samples [6]; some pathways associated with cancer were also found in adjacent cells. According to a recent theory about tumorigenesis by Slaughter [44], field cancerization results from the expansion of pre-neoplastic cells in early tumorigenesis. Aberrantly methylated genes in adjacent cells would affect the progress of hepatocarcinogenesis and would be useful in early detection and risk assessment.

This study is the first report on the global methylation profiling of hepatocytes isolated from tissues. Even with the small sample size, the aberrant methylation profile of HHCs is distinct from that of normal hepatocytes and reveals potential biomarkers in the diagnosis and prognosis of HBHC. These novel methylated genes should be further analyzed in larger clinical samples to elucidate their clinical implication, and more samples with different etiological backgrounds, pathophysiological processes, and histological characteristics should be used in further studies.

## Materials and Methods

### Patient specimens

All samples in this study were taken from a surgical specimen, obtained through hepatectomy or liver transplantation. All the patients have signed an informed-consent. No prisoners or organs from prisoners were used in this study. All patients included in this study were undergoing treatment in the First Affiliated Hospital of Zhejiang University in 2009. All experimental protocols and study methods were approved by the Ethics Committee of the First Affiliated Hospital, School of Medicine, Zhejiang University.

A total of 13 patients with HCC and 2 patients with hepatic hemangioma were selected for analysis. All patients were male to avoid sex-based variation, and the mean age was 46.84 years old (58±5.99). All HCC patients were hepatitis B virus (HBV) surface antigen-positive without hepatitis C virus (HCV) infection and exhibited the same underlying cirrhosis etiology. Clinical information for these patients were summarized in Tables 6 and 7.

**Table 6:** Clinicppathological features for 3 HBHC patients for methylation profiling analysis.

Varible	Case 1	Case 2	Case 3
**Case number**	631	560	564
**Age (year)**	58	41	53
**Gender**	male	male	male
**ALT**	42	35	47
**HBV-DNA**	6.31×10^4^	2.01×10^4^	3.33×10^5^
**Cirrhosis**	yes	yes	yes
**Preoperative AFP level (ng/ml)**	12.1	2	>58344
**Histopathologic grading**	moderate	moderate	poor
**Tumor size (cm)**	5	2	9
**Tumor number**	single	single	multiple
**PVTT**	0	0	1
**Childpagh**	B	B	C

**Table 7:** Clinicppathological features for 10 HBHC patients for validation analysis.

Variable		No. of cases
**No. of patients**		10
**Age(year)**	≤50	7
	>50	3
**Gender**	Male	10
	Female	0
**Preoperative AFP level(ng/ml)**	≤400	1
	>400	9
**Etiology**	HBV	10
	HCV+other	0
**Cirrhosis**	Yes	10
	No	0
**Tumor size(cm)**	≤50	3
	>50	7
**Tumor number**	single	4
	Multiple	6
**Childpagh**	A	4
	B	3
	C	3
**Histopathologic grading**	well+moderate	4
	poor	6
**PVTT**	Yes	3
	No	7

HBV surface antigen and anti-HCV antibody were measured serologically. The collection of tumor tissues was strictly limited inside the boundary of tumor, and adjacent non-cancerous liver tissues was collected at least 3 cm from the tumor margins, all the collections were under the supervision of a same pathologist. Each sample was devided into two parts, one was used for methylation profiling analysis, the other was histologically examined by two experienced pathologists, confirming that under the microscope there was no detectable normal liver cells inside the tumor samples and no tumor cells in the surrounding liver tissues.

Three paired HBHC and adjacent liver tissues were perfused to harvest single hepatocytes from tissues, and were then used for the Illumina Infinium BeadChips analysis. Ten paired HBHC and matching tissues were available for further validation. Two normal adult liver tissues from the patients with hepatic hemangioma who underwent partial hepatectomy without HBV or HCV infection were used for comparison purposes. Tissue samples were freshly frozen at −80°C or perfused immediately to harvest single cells.

### Isolation of hepatocytes from liver tissues

Three paired tumors and adjacent liver tissues and normal livers were isolated using a modified four-step collagenase perfusion method as described previously [30], [31]. The liver segments were perfused by the extracorporeal circulating perfusion apparatus in a sterile cell room. Four different buffer solutions supplemented, respectively, with 0.37 mg/ml EDTA, 0.5% dispase, 0.05% collagenase type IV (Gibco BRL, Grand Island, NY), and 40 ug/ml DNase I (Sigma, St. Louis, MO) were perfused successively into small hepatic vessels for 40 minutes. Solid tumor remnants were dispersed mechanically with a scalpel blade, and then incubated in collagenase buffer for 20 min at 37°C. The pooled suspensions were filtered by 100 um nylon mesh. Isolated cells were harvested and centrifuged at 4°C at 50 g for 3 minutes, and then the liver cells were resuspended in Dulbecco's modified Eagle's medium (DMEM; Invitrogen, Carlsbad, CA) and centrifuged twice at 4°C at 50 g for 1 min.

The hepatocytes were then stained using 0.4% trypan blue, and cell motility and the number of isolated cells were calculated. DNA was extracted from isolated hepatocytes and frozen liver tissues for methylation analysis using the QIAamp DNA Mini Kit (Qiagen, Valencia, Ca) according to the manufacturer's instructions.

### Illumina Infinium methylation assay

The Illumina Infinium 27k Human DNA methylation BeadChip v1.2 was used to obtain genome-wide DNA methylation profiles across approximately 27,000 CpGs, which were focused on the promoter regions of 14,495 genes, in purified cell samples.

Bisulfite conversion of genomic DNA was performed using the EZ DNA methylation Kit (Zymo Research, D5002, USA) according to the protocol provided by the Illumina Infinium Methylation Assay. Bisulfite-converted and -unconverted (i.e., methylated) sites were simultaneously evaluated using DNA hybridization to site-specific probes attached to beads (one set for unmethylated and the other for methylated sites), followed by allele-specific base extension that included a fluorescent label. Two different labels were used, and fluorescent signals were specific for either the T (unmethylated) or C (methylated) alleles. Methylation scores represented as β values were generated for each site using Illumina®Genome Studio software v1.0 (Illumina Inc., USA) and were computed based on the ratio of methylated to methylated plus unmethylated signal outputs. Thus, the β values ranged from 0 (unmethylated) to 1 (fully methylated) on a continuous scale. Initial array results were visualized using Illumina®Genome Studio software v1.0 (Illumina Inc., USA).

All computations and statistical analyses were performed using the R package (R v2.10.1; http://www.r-project.org) and Bioconductor [45]. Significant differences between the sample groups were identified using the limma (Linear Models for Microarray Analysis) package of the Bioconductor suite, and an empirical Bayesian-moderated t-statistic hypothesis test between the two specified phenotypic groups was performed [46] using significance at P value <0.05 with the absolute delta β value cutoff set at 0.15.

Heat maps were created using Mev software [47]. The Euclidean distance between two groups of samples was calculated by the average linkage measure (the mean of all pair-wise distances (linkages) between members of the two groups concerned). Gene annotation and enrichment analyses were performed using KEGG databases using the DAVID Bioinformatics Resources (2008; http://david.abcc.ncifcrf.gov/) [48] interfaces and WebGestalt (http://bioinfo.vanderbilt.edu/webgestalt/) [49], respectively.

### Combined bisulfite restriction analysis (COBRA) and bisulfite sequencing

Genomic DNA (1 µg) from freshly purified hepatocytes and liver tissues was bisulfite-converted using EpiTect Bisulfite Kits (Qiagen, Valencia, CA, USA) and used for PCR amplification. PCR primers were designed using Methyl Primer Express to amplify regions of CpG islands that overlapped or were close to the transcription start sites. All primer sequences and the mythylated sites are listed in Table 8. The PCR products were then digested with the methylation-sensitive endonuclease BstUI at 60°C for 2 hours. Digested PCR fragments were visualized using a 2% agarose gel, and the PCR products for bisulfite sequencing were cloned into the pGEM-T easy vector (Promage, USA) following the manufacturer's instructions. After being transfected into Escherichia coli, up to 10 clones were selected and sequenced using M13 reverse primers. The methylation status of each allele was determined using the methylation index (MI), which was calculated as the number of methylated CpG dinucleotides divided by the total number of CpG dinucleotides sequenced multiplied by 100.

**Table 8:** Primers used for COBRA and sequencing.

			Primer sequence		Position(TSS = +1)		
Offical symbol	Offical full name	Location	Forward	Reverse	Forward 5′	Reverse 5′	Length
**WNK2**	Wnk lysine deficient protein kinase 2	9q22.3	TTTTGGTTTATAGAGATGGA	CTTACAAAACAAAAACACCC	−20	+240	255 bp
**EMILIN2**	Elastin microfibril interfacer 2	18p11.3	TATTGGAAATGTTGTAACGTAGATT	ACCGTCTAAACTACCACATCC	−250	+164	414 bp
**TLX3**	T-cell leukemia homeobox 3	5q35.1	GAGTTAATGAGAGGAGAGAGGTT	AAAACCCTCCCAAATAACT	−157	+220	377 bp
**FOXE3**	forkhead box E3	1p32	TTAAATATAGTGGGATGGGGTT	CTCTAACTCCCGCCCTAA	−60	+354	414 bp
**GUCY1A2**	guanylate cyclase 1, soluble, alpha 2	11q21-q22	TTTTTGTTTGGGTCGTAGTT	TCTTCCTTCGAAACATACTACC	−88	+326	414 bp
**HIST1H4F**	histone cluster 1, H4f	6p21.3	TAAGGGGTTTTAGTGTGTAGTAAA	AATAAAACTCAACCACCAAAAC	−130	+250	380 bp
**OVOL1**	ovo-like 1(Drosophila)	11q13	GTAAGGGATTTCGTTTTTAGGG	CTCCAATTCCTCTTACACGTAA	−40	+277	277 bp
**TM6SF1**	transmembrane 6 superfamily member 1	15q24-q26	TTGGTAGGAGTATTGTAAGGACGT	CTCACTCACTCATACTAAACCGC	−214	+152	366 bp
**TRIM58**	tripartite motif-containing 58	1q44	GTTTGAGTGGTGGTTTTTTA	TAACCGACTCCTACAAAAAAT	−181	+72	253 bp
**ADAM8**	Adam metallopeptidase domain 8	10q26.3	TTTTATTTGTGTAAGGGAGGATG	ACCCAAAAACCACTATACACCT	−142	+287	429 bp
**KIF17**	kinesin family member 17	1p36.12	GGGGTTAGTTTTGGTTTCGT	CATACCCTACCGCCTACAAA	−24	+299	312 bp
**GRASP**	GRP1 (general receptor for phosphoinositides 1)-associated scaffold protein	12q13.13	TTTCGGAGTTTATAAAGGGAG	CGACAAAATAATAATCCTCCAAC	−142	+171	313 bp
**UTF1**	Undifferentiated embryonic cell transcription factor 1	10q26	GTTAGGATCGATTTTTTAGGA	CCAACAACAACTCCGTCT	−232	+174	406 bp
**ATP8A2**	ATPase, aminophospholipid transporter, class I, type 8A, member 2	13q12	TTCGGTTTTGCGTAATATTG	CATCTTAAAAACTTTATCCAAACCT	−238	+135	373 bp
**ADCY5**	adenylate cyclase 5	3q13.2-q21	GGGGTGTTAAGATGTTTCGT	AAACTAAAACCGAAACCCC	−226	+220	446 bp
**EPHA4**	EPH receptor A4	2q36.1	TTGGTAATGTTTTTAGTTCGTT	ACCATTCACACTAAACTCCC	−106	+187	293 bp
**BOLL**	bol, boule-like (Drosophila)	2q33	CGTTAGGTGGTAGGTTTAGGG	CCAAACCCTCAAAAACAAAC	−25	+258	283 bp

### Cell line culture conditions and 5-aza-dc treatment

Four hepatoma cell lines, HepG2, 7402, 97L and 97H, which were all routinely cultured in our institution, were grown in DMEM supplemented with 10% FBS at 37°C with humidified 5% CO2. Cells were seeded in six-well plates 24 hours before treatment with 10 µM 5-aza-2′-deoxycytidine (5-aza-dc; Sigma, USA) or phosphate-buffered saline as a control. Media and drugs were replaced every 24 h and the cells were harvested at 72 h post-treatment.

### Real-time quantitative polymerase chain reaction (RT-PCR) Analysis

Total RNA from the four hepatoma cell lines was extracted before and after treatment with 5-aza-dc using TRIzol (Invitrogen, Carlsbad, CA, USA) according to the manufacturer's protocols. RNA (1 µg) was reverse-transcribed using the AMV Reverse Transcription System(Invitrogen, Carlsbad, CA, USA). Primer sequences are listed in Table 9. RT-PCR was performed with SYBR Premix Ex TaqTM® (Takara, Japan) on an ABI prism 7500 sequencer. The relative expression levels were analyzed using the DDCt method, and GAPDH was used as an internal control.

**Table 9:** Reverse transcription-polymerase chain reaction (RT-PCR) primers.

Genes	Gene_ID	Primer sequences		Length	Anneal
**GRASP**	GeneID:160622	F	GGCGCTGGAGGACTATCAC	115 bp	60°C
		R	AGGACTCTGGCTGAGATTCTTC		
**HIST1H4F**	GeneID:8361	F	GCAAAGTGCTGCGTGACAAC	207 bp	60°C
		R	AGACAACATCCATTGCAGTGAC		
**TM6SF1**	GeneID:53346	F	CATCCCGGTCACCTATGTCTT	100 bp	60°C
		R	CAGCAGTGCTACCAGGAACA		
**TRIM58**	GeneID:25893	F	ACTTCAGCTCAGCAACAGCA	178 bp	60°C
		R	TACTCCTGGGACTGGGACAC		
**WNK2**	GeneID:65268	F	ACGTCTATGCCTTTGGGATGT	178 bp	60°C
		R	GATCTCGTACCTTTCCTCCTTGT		
**EMILIN2**	GeneID:84034	F	CCCCAACTGGTACAGCACAAC	139 bp	60°C
		R	AGGGAAGACTGGAGGTCAAGAA		
**TLX3**	GeneID:30012	F	AAAAGAGCCTCAACGACTCCATCC	117 bp	60°C
		R	TGACAGCGGGAACCTTGGAACTA		

### Statistical analysis

Data were evaluated using Student's t-test for each pair using SPSS software v16.0. The level of significance for all statistical analyses was P<0.05.

## Supporting Information

Table S1 **List of differentially methylated genes in single hepatocyte derived from HBHC (P<0.001).**
(DOC)

## References

[1] Blumberg BS (2010). Primary and secondary prevention of liver cancer caused by HBV.. Front Biosci (Schol Ed).

[2] Shi YH, Ding WX, Zhou J, He JY, Xu Y (2008). Expression of X-linked inhibitor-of-apoptosis protein in hepatocellular carcinoma promotes metastasis and tumor recurrence.. Hepatology.

[3] Wu LM, Yang Z, Zhou L, Zhang F, Xie HY (2010). Identification of histone deacetylase 3 as a biomarker for tumor recurrence following liver transplantation in HBV-associated hepatocellular carcinoma.. PLoS One.

[4] Zhong S, Tang MW, Yeo W, Liu C, Lo YM (2002). Silencing of GSTP1 gene by CpG island DNA hypermethylation in HBV-associated hepatocellular carcinomas.. Clinical Cancer Research.

[5] Moribe T, Iizuka N, Miura T, Kimura N, Tamatsukuri S (2009). Methylation of multiple genes as molecular markers for diagnosis of a small, well-differentiated hepatocellular carcinoma.. International Journal of Cancer.

[6] Lou C, Du Z, Yang B, Gao Y, Wang Y (2009). Aberrant DNA methylation profile of hepatocellular carcinoma and surgically resected margin.. Cancer Sci.

[7] Li B, Liu W, Wang L, Li M, Wang J (2010). CpG island methylator phenotype associated with tumor recurrence in tumor-node-metastasis stage I hepatocellular carcinoma.. Ann Surg Oncol.

[8] Um TH, Kim H, Oh BK, Kim MS, Kim KS (2010). Aberrant CpG island hypermethylation in dysplastic nodules and early HCC of hepatitis B virus-related human multistep hepatocarcinogenesis.. Journal of Hepatology.

[9] Lim SO, Gu JM, Kim MS, Kim HS, Park YN (2008). Epigenetic changes induced by reactive oxygen species in hepatocellular carcinoma: methylation of the E-cadherin promoter.. Gastroenterology.

[10] Gao W, Kondo Y, Shen L, Shimizu Y, Sano T (2008). Variable DNA methylation patterns associated with progression of disease in hepatocellular carcinomas.. Carcinogenesis.

[11] Deng YB, Nagae G, Midorikawa Y, Yagi K, Tsutsumi S (2010). Identification of genes preferentially methylated in hepatitis C virus-related hepatocellular carcinoma.. Cancer Sci.

[12] Archer KJ, Mas VR, Maluf DG, Fisher RA (2010). High-throughput assessment of CpG site methylation for distinguishing between HCV-cirrhosis and HCV-associated hepatocellular carcinoma.. Mol Genet Genomics.

[13] Lambert MP, Paliwal A, Vaissiere T, Chemin I, Zoulim F (2010). Aberrant DNA methylation distinguishes hepatocellular carcinoma associated with HBV and HCV infection and alcohol intake.. Journal of Hepatology.

[14] Bhowmick NA, Neilson EG, Moses HL (2004). Stromal fibroblasts in cancer initiation and progression.. Nature.

[15] Harrell JC, Dye WW, Harvell DM, Sartorius CA, Horwitz KB (2008). Contaminating cells alter gene signatures in whole organ versus laser capture microdissected tumors: a comparison of experimental breast cancers and their lymph node metastases.. Clin Exp Metastasis.

[16] Waanders LF, Chwalek K, Monetti M, Kumar C, Lammert E (2009). Quantitative proteomic analysis of single pancreatic islets.. Proc Natl Acad Sci U S A.

[17] Hanson JA, Gillespie JW, Grover A, Tangrea MA, Chuaqui RF (2006). Gene promoter methylation in prostate tumor-associated stromal cells.. J Natl Cancer Inst.

[18] Ai J, Tan Y, Ying W, Hong Y, Liu S (2006). Proteome analysis of hepatocellular carcinoma by laser capture microdissection.. Proteomics.

[19] Guedj N, Dargere D, Degos F, Janneau JL, Vidaud D (2006). Global proteomic analysis of microdissected cirrhotic nodules reveals significant biomarkers associated with clonal expansion.. Laboratory Investigation.

[20] Rescan PY, Clement B, Yamada Y, Segui-Real B, Baffet G (1990). Differential expression of laminin chains and receptor (LBP-32) in fetal and neoplastic hepatocytes compared to normal adult hepatocytes in vivo and in culture.. American Journal of Pathology.

[21] Lee CF, Ling ZQ, Zhao T, Fang SH, Chang WC (2009). Genomic-wide analysis of lymphatic metastasis-associated genes in human hepatocellular carcinoma.. World J Gastroenterol.

[22] Galli A, Ceni E, Mello T, Polvani S, Tarocchi M (2010). Thiazolidinediones inhibit hepatocarcinogenesis in hepatitis B virus-transgenic mice by peroxisome proliferator-activated receptor gamma-independent regulation of nucleophosmin.. Hepatology.

[23] Jeong JS, Lee SH, Jung KJ, Choi YC, Park WY (2003). Hepatotoxin N-nitrosomorpholine-induced carcinogenesis in rat liver: ex vivo exploration of preneoplastic and neoplastic hepatocytes.. Exp Mol Pathol.

[24] Yamaji S, Zhang M, Zhang J, Endo Y, Bibikova E (2010). Hepatocyte-specific deletion of DDB1 induces liver regeneration and tumorigenesis.. Proc Natl Acad Sci U S A.

[25] Hernandez-Vargas H, Lambert MP, Le Calvez-Kelm F, Gouysse G, McKay-Chopin S (2010). Hepatocellular carcinoma displays distinct DNA methylation signatures with potential as clinical predictors.. PLoS One.

[26] Tischoff I, Markwarth A, Witzigmann H, Uhlmann D, Hauss J (2005). Allele loss and epigenetic inactivation of 3p21.3 in malignant liver tumors.. International Journal of Cancer.

[27] Yuan Y, Wang J, Li J, Wang L, Li M (2006). Frequent epigenetic inactivation of spleen tyrosine kinase gene in human hepatocellular carcinoma.. Clinical Cancer Research.

[28] Calvisi DF, Ladu S, Conner EA, Seo D, Hsieh JT (2011). Inactivation of Ras GTPase-activating proteins promotes unrestrained activity of wild-type Ras in human liver cancer.. Journal of Hepatology.

[29] Qiu GH, Salto-Tellez M, Ross JA, Yeo W, Cui Y (2008). The tumor suppressor gene DLEC1 is frequently silenced by DNA methylation in hepatocellular carcinoma and induces G1 arrest in cell cycle.. Journal of Hepatology.

[30] Li J, Tao R, Wu W, Cao H, Xin J (2010). Transcriptional profiling and hepatogenic potential of acute hepatic failure-derived bone marrow mesenchymal stem cells.. Differentiation.

[31] Li J, Li L, Yu H, Cao H, Gao C (2006). Growth and metabolism of human hepatocytes on biomodified collagen poly(lactic-co-glycolic acid) three-dimensional scaffold.. ASAIO J.

[32] Yang ZF, Lau CK, Ngai P, Lam SP, Ho DW (2008). Cardiotrophin-1 enhances regeneration of cirrhotic liver remnant after hepatectomy through promotion of angiogenesis and cell proliferation.. Liver Int.

[33] Gerlyng P, Grotmol T, Erikstein B, Stokke T, Seglen PO (1992). Reduced proliferative activity of polyploid cells in primary hepatocellular carcinoma.. Carcinogenesis.

[34] Smiraglia DJ, Rush LJ, Fruhwald MC, Dai Z, Held WA (2001). Excessive CpG island hypermethylation in cancer cell lines versus primary human malignancies.. Human Molecular Genetics.

[35] Ma XD, Sui YF, Wang WL (2000). Expression of gap junction genes connexin 32, connexin 43 and their proteins in hepatocellular carcinoma and normal liver tissues.. World J Gastroenterol.

[36] Cronier L, Crespin S, Strale PO, Defamie N, Mesnil M (2009). Gap junctions and cancer: new functions for an old story.. Antioxid Redox Signal.

[37] Zhang W, Couldwell WT, Song H, Takano T, Lin JH (2000). Tamoxifen-induced enhancement of calcium signaling in glioma and MCF-7 breast cancer cells.. Cancer Research.

[38] Capiod T, Shuba Y, Skryma R, Prevarskaya N (2007). Calcium signalling and cancer cell growth.. Subcell Biochem.

[39] Fabregat I (2009). Dysregulation of apoptosis in hepatocellular carcinoma cells.. World J Gastroenterol.

[40] Ljunggren HG, Malmberg KJ (2007). Prospects for the use of NK cells in immunotherapy of human cancer.. Nat Rev Immunol.

[41] Hong C, Moorefield KS, Jun P, Aldape KD, Kharbanda S (2007). Epigenome scans and cancer genome sequencing converge on WNK2, a kinase-independent suppressor of cell growth.. Proc Natl Acad Sci U S A.

[42] Hill VK, Hesson LB, Dansranjavin T, Dallol A, Bieche I (2010). Identification of 5 novel genes methylated in breast and other epithelial cancers.. Mol Cancer.

[43] Wang Y, Yu Q, Cho AH, Rondeau G, Welsh J (2005). Survey of differentially methylated promoters in prostate cancer cell lines.. Neoplasia.

[44] Slaughter DP, Southwick HW, Smejkal W (1953). Field cancerization in oral stratified squamous epithelium; clinical implications of multicentric origin.. Cancer.

[45] Gentleman RC, Carey VJ, Bates DM, Bolstad B, Dettling M (2004). Bioconductor: open software development for computational biology and bioinformatics.. Genome Biol.

[46] Smyth GK (2004). Linear models and empirical bayes methods for assessing differential expression in microarray experiments.. Stat Appl Genet Mol Biol.

[47] Saeed AI, Sharov V, White J, Li J, Liang W (2003). TM4: a free, open-source system for microarray data management and analysis.. Biotechniques.

[48] Dennis G, Sherman BT, Hosack DA, Yang J, Gao W (2003). DAVID: Database for Annotation, Visualization, and Integrated Discovery.. Genome Biol.

[49] Zhang B, Kirov S, Snoddy J (2005). WebGestalt: an integrated system for exploring gene sets in various biological contexts.. Nucleic Acids Res.

