# Supplementary material for: Methylation Profile of Single Hepatocytes Derived from Hepatitis B Virus-Related Hepatocellular Carcinoma
Source: PLoS One. 2011 May 23;6(5):e19862. doi: 10.1371/journal.pone.0019862 (PMC3100314; doi:10.1371/journal.pone.0019862)
Supplement: Table S1 — List of differentially methylated genes in single hepatocyte derived from HBHC (P<0.001). (DOC) [file pone.0019862.s001.doc]

| **Table S1.** **List of differentially methylated genes in single hepatocyte derived from HBHC (P<0.001)** | | | | | | | | | |
| --- | --- | --- | --- | --- | --- | --- | --- | --- | --- |
|  |  |  |  |  |  | **hcc vs nl differential methylation** | | **hcc vs aj differential methylation** | |
| **probe ID** | **SYMBOL** | **CHR** | **GENE_ID** | **DISTANCE_TO_TSS** | **CPG_ISLAND** | **delta beta** | **P.Value** | **delta beta** | **P.Value** |
| cg20616414 | WNK2 | 9 | GeneID:65268 | 184 | TRUE | 0.543147886 | 4.16647E-06 | 0.4868135 | 4.11123E-06 |
| cg18815943 | FOXE3 | 1 | GeneID:2301 | 570 | TRUE | 0.542965619 | 9.8383E-06 | 0.5020897 | 7.95163E-06 |
| cg15408454 | MAGEA6 | X | GeneID:4105 | 18 | TRUE | -0.286929879 | 2.17401E-05 | -0.2981015 | 8.38994E-06 |
| cg23984434 | GUCY1A2 | 11 | GeneID:2977 | 515 | TRUE | 0.341534439 | 1.01458E-05 | 0.3132392 | 8.64322E-06 |
| cg09009111 | EMILIN2 | 18 | GeneID:84034 | 281 | TRUE | 0.395398779 | 1.54827E-05 | 0.3778466 | 1.01616E-05 |
| cg07533148 | TRIM58 | 1 | GeneID:25893 | 311 | TRUE | 0.449988829 | 3.70417E-05 | 0.4379473 | 2.17846E-05 |
| cg17886204 | DKFZp434I1020 | 15 | GeneID:196968 | 138 | TRUE | 0.376850156 | 3.2596E-05 | 0.3587906 | 2.20021E-05 |
| cg13878010 | ADCY5 | 3 | GeneID:111 | 116 | TRUE | 0.496224658 | 1.69316E-05 | 0.419856 | 2.40541E-05 |
| cg08766149 | GZMB | 14 | GeneID:3002 | 131 | FALSE | -0.38448613 | 2.91373E-05 | -0.3504511 | 2.58737E-05 |
| cg20909686 | OVOL1 | 11 | GeneID:5017 | 487 | TRUE | 0.424590061 | 1.40827E-05 | 0.3323605 | 3.26838E-05 |
| cg13993218 | INS | 11 | GeneID:3630 | 558 | FALSE | -0.284995755 | 0.000243326 | -0.34958 | 3.51522E-05 |
| cg16786703 | ADAM8 | 10 | GeneID:101 | 117 | TRUE | 0.587122457 | 2.96256E-05 | 0.500142 | 4.02121E-05 |
| cg15772361 | SERPINB3 | 18 | GeneID:6317 | 891 | FALSE | -0.230873231 | 8.69344E-05 | -0.2242818 | 5.21777E-05 |
| cg04034767 | GRASP | 12 | GeneID:160622 | 159 | TRUE | 0.630643854 | 4.48242E-05 | 0.5439968 | 5.61519E-05 |
| cg20881888 | ZMYND10 | 3 | GeneID:51364 | 77 | TRUE | 0.499525418 | 4.45196E-05 | 0.4297561 | 5.66942E-05 |
| cg09053680 | UTF1 | 10 | GeneID:8433 | 336 | TRUE | 0.628058797 | 0.000130007 | 0.6393068 | 5.87549E-05 |
| cg17560332 | BOLL | 2 | GeneID:66037 | 365 | TRUE | 0.460854829 | 4.71597E-05 | 0.3828796 | 7.45042E-05 |
| cg14696396 | TM6SF1 | 15 | GeneID:53346 | 66 | TRUE | 0.393190662 | 9.27701E-05 | 0.3620392 | 7.76085E-05 |
| cg07545232 | MAGEA3 | X | GeneID:4102 | 2 | TRUE | -0.277667901 | 0.000256899 | -0.3004448 | 8.10595E-05 |
| cg15613048 | KIF17 | 1 | GeneID:57576 | 479 | TRUE | 0.48254299 | 0.000133392 | 0.4669103 | 8.24752E-05 |
| cg14304761 | SYK | 9 | GeneID:6850 |  | TRUE | 0.481765391 | 7.28505E-05 | 0.41259 | 9.51544E-05 |
| cg03776464 | EPHA4 | 2 | GeneID:2043 | 63 | TRUE | 0.374194522 | 0.000159287 | 0.3564153 | 0.000108691 |
| cg18236477 | ATP8A2 | 13 | GeneID:51761 | 49 | TRUE | 0.508461976 | 7.65304E-05 | 0.428749 | 0.000109874 |
| cg08260959 | HIST1H4F | 6 | GeneID:8361 | 266 | TRUE | 0.299601097 | 0.000318792 | 0.3112636 | 0.000129876 |
| cg25720804 | TLX3 | 5 | GeneID:30012 | 101 | TRUE | 0.541610306 | 0.000165148 | 0.4960181 | 0.000143141 |
| cg14384532 | NTRK3 | 15 | GeneID:4916 | 963 | TRUE | 0.387492416 | 0.000164242 | 0.3534689 | 0.000145817 |
| cg07730329 | PCDHGA12 | 5 | GeneID:26025 | 21 | TRUE | 0.283555393 | 0.000219167 | 0.2684674 | 0.000155639 |
| cg24662718 | VAV3 | 1 | GeneID:10451 | 70 | TRUE | 0.509360165 | 0.000314406 | 0.5105597 | 0.000159111 |
| cg09736162 | CELSR3 | 3 | GeneID:1951 | 95 | TRUE | 0.379611681 | 0.00010024 | 0.3146894 | 0.000159154 |
| cg21460081 | HOXB4 | 17 | GeneID:3214 | 269 | TRUE | 0.359902115 | 0.000182244 | 0.3288881 | 0.000160122 |
| cg20395892 | IRAK3 | 12 | GeneID:11213 | 237 | TRUE | 0.250043749 | 0.000372732 | 0.2560159 | 0.000166627 |
| cg20085077 | ARMCX4 | X | GeneID:158947 | 41 | TRUE | 0.465169413 | 0.000101164 | 0.3823356 | 0.000169099 |
| cg00143998 | HIST3H2BB | 1 | GeneID:128312 | 51 | TRUE | 0.270856761 | 0.000355877 | 0.2736068 | 0.000172379 |
| cg00463577 | C6orf150 | 6 | GeneID:115004 | 88 | TRUE | 0.535251914 | 0.000264775 | 0.5135558 | 0.000173921 |
| cg01519742 | JAKMIP1 | 4 | GeneID:152789 | 191 | TRUE | 0.567228158 | 0.000180932 | 0.4865536 | 0.000232534 |
| cg13274254 | GULP1 | 2 | GeneID:51454 | 176 | TRUE | 0.262574706 | 0.000181229 | 0.2234835 | 0.000243984 |
| cg03958979 | NR2E1 | 6 | GeneID:7101 | 875 | TRUE | 0.411041954 | 0.000154026 | 0.3370599 | 0.000259402 |
| cg21518208 | KRTHB5 | 12 | GeneID:3891 | 107 | FALSE | -0.341610529 | 0.000315836 | -0.3097371 | 0.000291376 |
| cg11452221 | GEFT | 12 | GeneID:115557 | 388 | TRUE | 0.315333493 | 0.000271134 | 0.2756964 | 0.000310283 |
| cg22836229 | EFCAB1 | 8 | GeneID:79645 | 74 | TRUE | 0.458549443 | 0.000221404 | 0.386007 | 0.000317503 |
| cg10938286 | CST2 | 20 | GeneID:1470 | 313 | FALSE | -0.294267411 | 0.000697221 | -0.3000577 | 0.000325616 |
| cg05158615 | NPY | 7 | GeneID:4852 | 250 | TRUE | 0.333195209 | 0.000461112 | 0.315651 | 0.000329251 |
| cg24432073 | CDKL2 | 4 | GeneID:8999 | 63 | TRUE | 0.436094161 | 0.000414667 | 0.4037843 | 0.00033847 |
| cg03421687 | ZMYND10 | 3 | GeneID:51364 | 95 | TRUE | 0.396402247 | 0.00030139 | 0.3424612 | 0.000369803 |
| cg26848126 | CYSLTR1 | X | GeneID:10800 | 174 | FALSE | -0.304041772 | 8.683E-05 | -0.2132839 | 0.00037446 |
| cg23753610 | DNAHL1 | 17 | GeneID:284176 | 426 | FALSE | -0.421924702 | 0.000727998 | -0.4185694 | 0.000400083 |
| cg12265829 | ADCY4 | 14 | GeneID:196883 | 158 | TRUE | 0.482406494 | 0.000398328 | 0.4303123 | 0.000404683 |
| cg16791508 | KRTHB3 | 12 | GeneID:3889 | 520 | FALSE | -0.458436554 | 0.000870824 | -0.4655092 | 0.000419922 |
| cg18342279 | ZAR1 | 4 | GeneID:326340 | 35 | TRUE | 0.460444045 | 0.000485095 | 0.4204837 | 0.000429802 |
| cg16542081 | GATA5 | 20 | GeneID:140628 |  | TRUE | -0.27555998 | 0.000580865 | -0.2570007 | 0.000455838 |
| cg19355190 | EGR2 | 10 | GeneID:1959 | 329 | TRUE | 0.328727819 | 0.000141104 | 0.2388985 | 0.000484654 |
| cg21991396 | CIAS1 | 1 | GeneID:114548 | 66 | FALSE | -0.326136493 | 0.00011107 | -0.2272107 | 0.000492931 |
| cg03734874 | FLJ42486 | 14 | GeneID:388021 | 285 | TRUE | 0.446449154 | 0.000589066 | 0.4095389 | 0.000509035 |
| cg00290506 | CNIH3 | 1 | GeneID:149111 | 47 | TRUE | 0.357295638 | 0.000944218 | 0.3535056 | 0.000530883 |
| cg22218909 | DEFA3 | 8 | GeneID:1668 | 570 | FALSE | -0.276516005 | 0.00078539 | -0.2613329 | 0.000573004 |
| cg06825142 | DRD4 | 11 | GeneID:1815 | 135 | TRUE | 0.570457026 | 0.000876515 | 0.5412566 | 0.000626291 |
| cg05675373 | KCNC4 | 1 | GeneID:3749 | 280 | TRUE | 0.407513808 | 0.000786851 | 0.3779316 | 0.000639948 |
| cg00208967 | OLFM2 | 19 | GeneID:93145 | 854 | TRUE | 0.421346262 | 0.000605644 | 0.3706611 | 0.000666144 |
| cg00892798 | NGFR | 17 | GeneID:4804 | 242 | TRUE | 0.36264342 | 0.000627972 | 0.3183102 | 0.000699433 |
| cg05684891 | DAB2IP | 9 | GeneID:153090 |  | TRUE | 0.638350214 | 0.000322897 | 0.4963104 | 0.00072827 |
| cg20339230 | ST8SIA2 | 15 | GeneID:8128 | 220 | TRUE | 0.310849718 | 0.000973683 | 0.2844277 | 0.000856586 |
| cg15457899 | SCN3B | 11 | GeneID:55800 | 353 | TRUE | 0.4913728 | 0.000808516 | 0.4347449 | 0.000859843 |
| cg23881725 | DLEC1 | 3 | GeneID:9940 | 54 | TRUE | 0.390018872 | 0.000478083 | 0.3144814 | 0.000866302 |
